# Rebelling for a Reason: Protein Structural “Outliers”

**DOI:** 10.1371/journal.pone.0074416

**Published:** 2013-09-20

**Authors:** Gandhimathi Arumugam, Anu G. Nair, Sridhar Hariharaputran, Sowdhamini Ramanathan

**Affiliations:** National Centre for Biological Sciences, Tata Institute of Fundamental Research, Gandhi Krishi Vigyana Kendra Campus, Bangalore, India; MRC National Institute for Medical Research, United Kingdom

## Abstract

Analysis of structural variation in domain superfamilies can reveal constraints in protein evolution which aids protein structure prediction and classification. Structure-based sequence alignment of distantly related proteins, organized in PASS2 database, provides clues about structurally conserved regions among different functional families. Some superfamily members show large structural differences which are functionally relevant. This paper analyses the impact of structural divergence on function for multi-member superfamilies, selected from the PASS2 superfamily alignment database. Functional annotations within superfamilies, with structural outliers or ‘rebels’, are discussed in the context of structural variations. Overall, these data reinforce the idea that functional similarities cannot be extrapolated from mere structural conservation. The implication for fold-function prediction is that the functional annotations can only be inherited with very careful consideration, especially at low sequence identities.

## Introduction

The availability of protein three-dimensional structures repeatedly confirms that a limited number of folds are shared by large number of protein sequences. This limitation is imposed by the physical chemistry of the polypeptide [1]–[3]. Both large-scale genomic surveys and studies of individual superfamilies have demonstrated that protein structure is often conserved between evolutionarily related proteins, even at undetectable sequence similarity [4]. According to SCOP [5], protein domains are grouped into the same fold, if they have the same major secondary structure elements with same orientation and topological connections. The next level of classification of proteins is superfamily; which is a level defined to contain one or more families with protein domains thought to have common evolutionary origin [6].

Currently, it is quite uncommon to discover a new fold, while it is possible to observe a subtle conformational difference arising from some very common structural motifs [7]. The presence of such structural differences can be attributed to various reasons such as addition/deletion, circular permutation, strand inversion or withdrawal and β-hairpin flip/swap [8]. Several groups have already investigated the structural features, both similarities and divergence, in various superfamilies [9]–[11]. Structural variation across domains in superfamilies has also been examined by other groups [12], [13]. The extent to which structural domain classifications help us to understand the relationship between sequence and structure of a protein to its function has also been a focus in the past [14]. A vast amount of literature already exists on the enzyme superfamilies with diverse functions [15], [16]. Usually, a difference in Enzyme Commission (E.C.) number [17] is reflected by either subtle or obvious differences in function.

Analysis of protein domains at the superfamily level is biologically significant to study the association of evolutionary, functional and structural perspectives of domains. Structure alignment is the method of choice for comparing the superfamily members of minimal sequence identity [18]. Structural deviations of protein structures are generally measured by root mean square deviation (RMSD), which provides a measure of the average distance between aligned C^α^ atoms of superimposed proteins. There is an increasing evidence that, in some superfamilies, domains have undergone significant structural changes during evolution [19], [20]. Such superfamilies with members of high conformational variability will become a challenge for any structure alignment program. Recent structure alignment programs started giving emphasis on structure flexibility while aligning the protein structures. This may increase the alignment consistency but it will not address the intrinsic ambiguity arising due to structural divergence that could reside even in the structural core [21]. Many structure alignment programs usually focus on optimizing the geometrical similarities without considering structural features such as secondary structures, hydrogen bonding and solvent accessibility [22].

PASS2 [23] is a structure alignment database of distantly related protein domains (less than 40% pairwise sequence identity) which directly corresponds to SCOP. The PASS2 database contains superfamily members with less than 40% sequence identity which are considered as representative set of distantly related protein domains. The automated version of CAMPASS is called as PASS2 [24], which we now refer to as PASS2.1, contain 613 superfamilies in direct correspondence with SCOP 1.53. The subsequent versions of PASS2.2 and PASS2.3 [25], [26] have been created and updated in direct correspondence with SCOP1.63 and SCOP 1.73, respectively. All these versions differ in the superfamily dataset used and also with respect to the improvement of the alignment protocol with minimal manual intervention. A good structural alignment at the superfamily level is of high importance in structure modeling exercises *i.e.*, threading a sequence to a framework structure, derived from common structural feature of a superfamily [27]. After comparing the structures, we found that around 80% of the multi-member superfamilies have a highly conserved structural core which is reflected by very low RMSD after superposition. However, 20% of multi-member superfamilies have domains with high structural variations and these domains are termed as ‘structurally deviant member’ or ‘outlier’ of the superfamily. These structural differences of a member within a superfamily can occur due to repetitions, deletion, insertion, circular permutations and considerable conformational variability. Interestingly, in some superfamilies, these deviant members belong to one particular family implying that they are functionally also distinct and diverse.

In this paper, we are mainly focusing on multi-member superfamilies which exhibit one or two structurally deviant members. We show that, it is possible to employ structure alignment protocol (http://www.nature.com/protocolexchange/protocols/604) to identify the structurally deviant members with family-specific functional differences within a superfamily. The aim of this paper is to provide a detailed description of functional variations of outliers in protein domain superfamilies and to illustrate that the structural divergence is found in certain domains which may be related at the superfamily level. Sometimes, large structural differences are introduced with a functional importance.

## Methods

### Structure-based Sequence Alignment of Superfamily Domains

PASS2 [28] database contains structure-based sequence alignment of protein domain superfamilies in correspondence with SCOP 1.75. A PASS2 superfamily is a subset of corresponding SCOP superfamily, with no member sharing more than 40% sequence identity with any of the other members. We have mainly focused on multi-member superfamily (MMS; which implies multiple number of superfamily members) with <40% identity with other domains in the superfamily.

### Alignment Procedure

The structural alignment of multi-member superfamilies is performed using the standard protocol of PASS2. The initial alignment is performed using MATT [29] program, where short structural fragments from all the proteins are aligned against each other optimally and the final alignment brings these together in geometrically consistent ways. The initial equivalences, derived from the aligned positions and a structure-guided tree are typical inputs for the program COMPARER [27]. COMPARER alignment procedure uses variable gap penalties, local structural features such as backbone conformation, solvent accessibility and hydrogen bonding patterns. In general, the variable gap penalties ensure that there are no unreasonable gaps in between secondary structures and conserved regions within the alignment. After the final alignment through COMPARER, JOY program is employed to recognize all non-gap alignment positions as equivalences. Such equivalences are employed for rigid-body superposition using MNYFIT [30]. MNYFIT is used to obtain superimposed structures, through Euclidean transformations. The pairwise RMSDs, obtained from matched C^α^s, are utilized by the in-house developed program MeanRMSD. The program provides average of one-against-all RMSD measure for each member in the superfamily. A high Mean RMSD value for a member indicates significant variations in the structure of the member with respect to other members within a superfamily. A threshold of 5.5Å was set after a careful analysis within the superfamily alignments, obtained earlier, by a careful manual alignment [23] and used in our earlier analyses (http://www.nature.com/protocolexchange/protocols/604). These outliers are also verified by TMSCORE [31] which is used for similarity measurement between two structures. In general, all the outliers have a TMSCORE less than 0.5, which corresponds to significant structural difference. A superfamily member can have a variation in the structural core, with high RMSD and low TMSCORE (thresholds defined above), due to change in number of secondary structural elements, architecture, topology or any of their combinations. These members are termed as ‘structurally deviant members’ of the superfamily.

### Functional Similarity Based on GO - Terms

Functional similarity of gene products could be estimated by controlled biological vocabularies, such as Gene Ontology (GO) [32]. A quantitative comparison of functional similarity is more informative for understanding the biological role and function of genes [33]. Semantic similarity is a quantitative assessment of relatedness or similarity of function between two protein domains. Higher semantic score implies that the domains are functionally more similar. Individual semantics value is calculated between two GO-terms using G-SESAME [34]. For instance, if two domains, d1eu1a1 and d2iv2x1 of ADC-superfamily are described by GO terms, molybdenum ion binding (GO:0030151) and formate dehydrogenase (NAD+) activity (GO:0008863), respectively, their GO semantic similarity is 0.077 as per G-SESAME calculations. Mean semantics similarity attributed to a pair of domains in a superfamily is the average of all possible GO terms that could be compared across the two domains. If two domains, have more than one GO term descriptors, say 1eu1a1 and 1kqfa1 of ADC superfamily i.e. GO:0030151 : molybdenum ion binding for 1eu1a1 and GO:0008863:formate dehydrogenase activity, GO:0046872:metal ion binding for 1kqfa1, their mean semantics similarity is the average of 0.077 and 0.754 (which is 0.4155). However, for a PASS2 superfamily consisting of multiple members, the GO annotations are compared for all possible pairs of domains and hence the GO semantics value attributed to a domain, like 1eu1a1, is the grand average of all possible pairwise mean-semantics-similarity involving a domain of interest. GO semantics similarity value for outliers and non-outliers can be compared for a superfamily that harbours few members as outliers.

## Results and Discussion

### Structurally Deviant Members of PASS2

**Figure 1:**
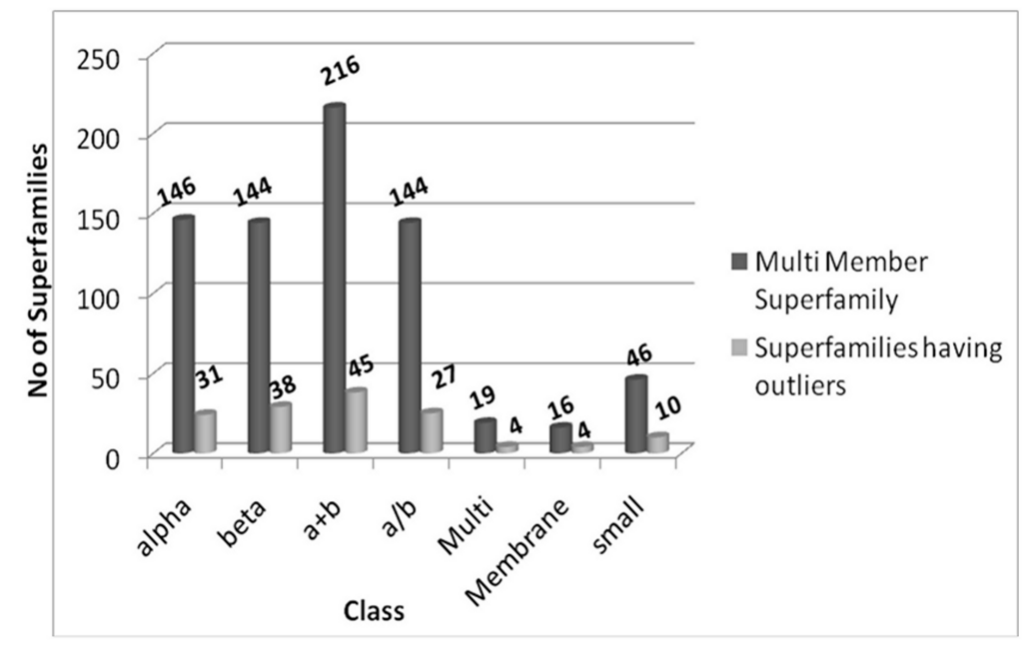
Total number of multi-member superfamilies and superfamilies having structurally deviant domains according to structural class. (Please see Methods for definition of ‘structural deviants’).

Here, we emphasize that using an appropriate structure alignment protocol even on protein domains with low sequence identity, one can identify structural differences which occur due to a functional reason. After the structural alignment of 731 multi-membered superfamilies, 159 superfamilies show one or more structurally deviant members within the superfamily. Figure 1 shows the total multi-member superfamilies and superfamilies having outliers, grouped according to structural class. These outliers generally exhibit high RMSD >5.5 and they are again confirmed by visual inspection.

**Figure 2:**
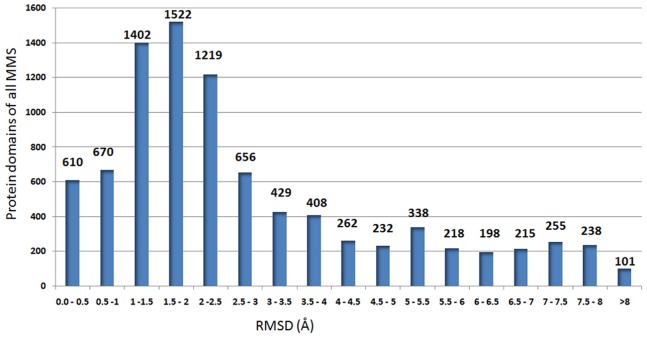
Mean RMSD plot for 8973 members of 731 multi-membered superfamilies. Outlier protein domains are with RMSD greater than 5.5 Å. (Please see Methods definition of structure deviants).

These 159 superfamilies are characterized as single, two and multiple-outlier superfamilies (Figure S1) (for the full list of superfamilies, please see http://caps.ncbs.res.in/download/pass2_outliers/outliers_tables/). 41 superfamilies from the category of single and two outlier superfamilies are highly interesting, since they retain outliers which are family-specific in nature suggesting a functional context. Table 1 summarizes the details of all 41 superfamilies with the structural reasons caused for the family-specific functional implications of the outliers. Superfamilies with multiple outliers may form subgroups and cluster occasionally (for example, see Figure S2). The other superfamilies have major structural embellishments which contribute to high RMSD and become harder and diverse to consolidate for discussions (for the spread of RMSD, please see Figure 2).

**Table 1:** Details of all family-specific outliers in PASS2^a^ multi-member superfamilies.

S.NO	SCOPCode	Superfamily@	Number ofPASS2^a^members	Outliers	Outliers_family	Reasons
1	55103	FAD-linked oxidases, C-terminaldomain	5	d2i0ka1	Cholesterol oxidase	Structural and Conformational difference
2	81593	Nucleotidyl transferase substratebinding subunit/domain	5	d1v4aa1	HEPN domain	Structural and Conformational difference
3	49695	gamma-Crystallin-like	12	d1bhua_	Streptomyces metalloproteinaseinhibitor, SMPI	Conformational difference of secondary structures
4	57262	Leech antihemostatic proteins	5	d1bx7a_	Huristasin-like	Structural and Conformational difference
5	50447	Translation proteins	20	d1vqob1, d2gycb1	Ribosomal protein L3	Distinct topology and architecture
6	55909	Pentein	12	d1g61a_, d1g62a_	Ribosome anti-association factoreIF6	Distinct topology and architecture
7	56059	Glutathione synthetaseATP-binding domain-like	22	d1eucb2, d2nu7b2	Succinyl-CoA synthetase, beta-chain,N-terminal domain	Distinct architecture and topology
8	143437	THUMP domain-like	4	d1rkia1	PAE0736-like	Distinct architecture and topology
9	51219	TRAP-like	3	d1gtfa_	Trp RNA-binding attenuationprotein (TRAP)	Distinct architecture and topology
10	51246	Rudiment single hybrid motif	7	d1e2w2	Cytochrome f, small domain	Distinct architecture and topology
11	52799	(Phosphotyrosine protein)phosphatases II	20	d1ywfa1	Mycobacterial PtpB-like	Distinct architecture and topology
12	56747	Prim-pol domain	4	d1ro0a_	Bifunctional DNA primase/polymerase N-terminal domain	Distinct architecture and topology
13	56935	Porins	11	d1t16a_	Outer membrane proteintransport protein	Distinct architecture and topology
14	50692	ADC-like*	16	d1ppya_, d1uhez1	Pyruvoyl dependent aspartate decarboxylase, ADC	Distinct architecture and topology
15	52016	LeuD/IlvD-like	6	2gp4a1	IlvD/EDD C-terminal domain-like	Distinct topology and architecture
16	55088	Methyl-coenzyme M reductasesubunits	3	d1hbnc_	Methyl-coenzyme M reductasegamma chain	Distinct topology and architecture
17	48113	Heme-dependent peroxidases*	8	d1cxpz1, d1q4ga1	Myeloperoxidase-like	Deviation concentrated in specific taxa to different mode of substrate binding
18	51730	FAD-linked oxidoreductase*	3	d1tj1a2	Proline dehydrohenase domainof bifunctional PutA protein	Circular permutation and different EC number
19	56655	Carbohydrate phosphatase	8	d1ni9a_	GlpX-like bacterial fructose-1,6-bisphosphatase	Permuted topology
20	46548	Alpha-helical ferredoxin*	4	d1gtea1	Dihydropyrimidinedehydrogenase,N-terminal domain	Different EC number and structural elaborations with added secondary structural elements
21	110849	ParB/Sulfiredoxin	4	d1vz0a2	ParB-like nuclease domain	Difference in topology and secondary structure content
22	46934	UBA-like	30	d1mn3a_	CUE domain	Difference in topology and secondary structures
23	55144	LigT-like	4	d2ilxa1	2′,3′-cyclic nucleotide 3′-phosphodiesterase, catalytic domain	Difference in the structural topology
24	81631	PAP/OAS1 substrate-binding domain*	5	d2pbea1	AadK C-terminal domain-like	Difference in the architecture of the secondary structures.
25	48295	TrpR-like	4	d1jhga_	Trp repressor, TrpR	Difference in the topology of secondary structures
26	82615	Polo-box domain *	3	d1mbya_	Swapped Polo-box domain	Domain swapping
27	50715	Ribosomal protein L25-like	4	d1gtra1	Gln-tRNA synthetase (GlnRS),C-terminal (anticodon-binding)domain	Duplication, consists of two barrel domains with the swapping of N-terminal strands
28	56436	C-type lectin-like	36	d1t61a1, d1t61a2	Noncollagenous (NC1) domain ofcollagen IV	Segment swapping with subdomains of domain
29	82057	Prokaryotic SH3-related domain	5	d1xova1	Ply C-terminal domain-like	tandem repeat of two SH3-like domains swapped with the N-terminal strands
30	52266	SGNH hydrolase *	13	d1flca2	Esterase domain of haemagglutinin-esterase-fusion glycoprotein HEF1	Extension of N and C-terminal part
31	53649	Alkaline phosphatase-like	9	d1ei6a_	Phosphonoacetate hydrolase	Insertion of alpha+beta subdomain near C-terminus
32	55003	PAP/Archaeal CCA-adding enzyme, C-terminal domain	3	d1r89a3	Archaeal tRNA CCA-adding enzyme	Insertion of large part of secondary structures
33	55781	GAF domain-like	12	d1stza2	HrcA C-terminal domain-like	Insertion of a secondary structures
34	55154	CYTH-like phosphatases	5	d1d8ia_	mRNA triphosphatase CET1	Extra elaborations to the core
35	50939	Sialidases	11	d1v0ea1	Endo-alpha-sialidase	Outlier has extra N-terminal domain and insert domain
36	89095	GatB/YqeY motif	4	d1ng6a_	GatB/YqeY domain	Difference in secondary structure content due to insertion
37	56634	Heme-dependent catalase-like	4	d1u5ua_	Allene oxide synthase	Difference in secondary structural content and conformation
38	51679	Bacterial luciferase-like	7	d1nfpa_	Non-fluorescent flavoprotein (luxF, FP390)	Incomplete core structure of beta/alpha barrel with mixed beta-sheet of 7 strands
39	48317	Acid phosphatase/Vanadium-dependent haloperoxidase	4	d1vnsa_	Chloroperoxidase	Duplication of secondary structures
40	89957	MTH1187/YkoF-like*	6	d1s99a_	Putative thiamin/HMP-binding protein YkoF	Internal repeat or duplication
41	55205	EPT/RTPC-like	4	d1qmha2	RNA 3′-terminal phosphate cyclase, RPTC	Non-duplicated fold of *beta-alpha-beta-alpha-beta(2)*

a Please see http://caps.ncbs.res.in/pass2/for full alignments.

@The superfamilies marked with ‘*’ symbol are further considered for illustration. Also see http://caps.ncbs.res.in/download/pass2_outliers/FIR_map/for mapping of functionally important residues for each of these superfamilies.

All the 41 superfamilies with family-specific outliers are critically investigated for the nature of structural variations mainly by visual inspection and often confirmed by SCOP records (Table 1). The study provides information about some of the important structural reasons for this functional diversity. The reasons could be due to simple difference in the structure and conformation as the core structure remains intact (four superfamilies), distinct architecture and topology leads to different core structure and functional variation (12 superfamilies), structural deviation in specific taxa leads to different mode of substrate binding (one superfamily), circular permutation where the protein structure connectivity is altered (two superfamilies), mechanistically diverse enzyme families with obvious functional difference at domain linker regions (one superfamily), differences in the secondary structural elements and topology (five superfamilies), structural divergence exist between swapped and non-swapped domain/segment (four superfamilies), insertion of secondary structures which leads to structural embellishments (seven superfamilies), deletion of secondary structures that could lead to incomplete and disordered core structures (two superfamilies), duplication/non-duplication of small domain or set of secondary structures (two superfamilies). (For detailed structural explanation of all these superfamilies, please see http://caps.ncbs.res.in/download/pass2_outliers/functional_outliers). We discuss these reasons more elaborately using one illustrative superfamily each (*-mark in Table 1 for illustrative superfamilies) and details are provided for all the 41 superfamilies.

### Family-specific Domains with Distinct Topology and Architecture Leads to Functional Variation

There are total of 12 superfamilies which exhibit difference in the topology and architecture which leads to change in the core structure. These superfamilies are translation proteins, penteins, Glutathione synthetase ATP-binding-like domains, THUMP-like domains, TRAP-like domains, Rudiment single hybrid motifs, (Phosphotyrosine protein) phosphatases II, Prim-pol domains, Porins, ADC-like, LeuD/IlvD-like, Methyl-coenzyme M reductase subunit. Since larger number of superfamilies fall into this category of family specific architecture and topology, perhaps these major structural changes could ultimately lead to the functional diversity of the domains. An additional four superfamilies (C-terminal domain of FAD-linked oxidases, Nucleotidyl transferase substrate binding subunit/domain, gamma-crystallin-like and leech antihemostatic proteins) have domains with family-specific difference in the conformations of secondary structures, but, they did not show any topological difference.

**Figure 3:**
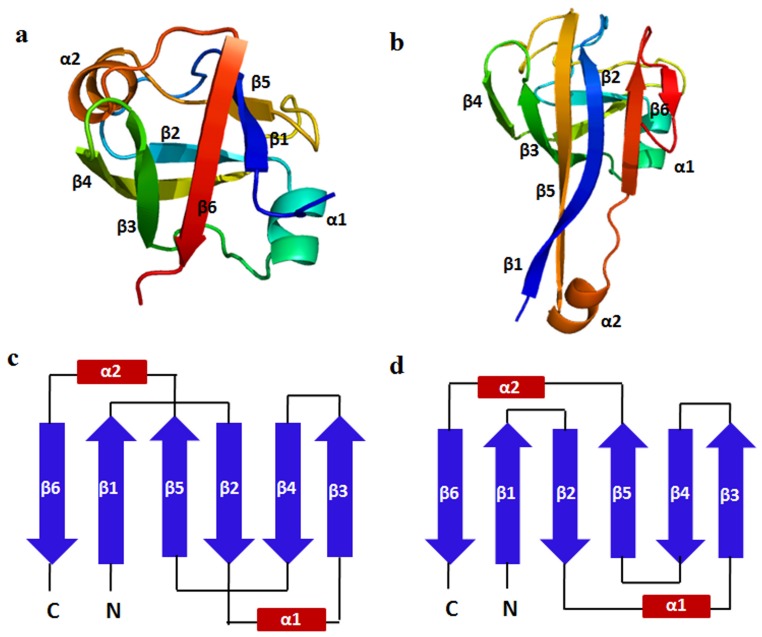
Topological differences seen in ADC-like superfamily. (a) A representative structure of the ADC like superfamily that has ferredoxin reductase-like topology (b) Double psi-β-barrel fold observed in Pyruvoyl dependent aspartate decarboxylase (ADC) family. (c) Secondary structure arrangement and topological connections observed in ferredoxin reductase fold. (d) Arrangements as seen in double psi-β-barrel fold.

ADC-like superfamily consists of 16 domains with the topology of β-barrel with cross-over loops. All these 16 domains contain six β-strands and two α-helices. Using structure-based sequence alignment, two domains have been observed as outliers with interesting topological differences and they belong to pyruvoyl dependent aspartate decarboxylase family (ADC). ADC is an unusual enzyme, as its catalysis depends on the pyruvoyl group formed as a result of self-processing [35]. ADC family proteins are generally involved in catalyzing the conversion of L-aspartate to β-alanine and provide the major route of β-alanine production which is essential for the biosynthesis of pantothenate (Vitamin B_5_). ADC is observed to be present in bacteria, fungi and plants [36]. The remaining 14 domains include C-terminal domain of formate dehydrogenase/DMSO reductases and N-terminal domain of Cdc48 domain-like family. The former plays a crucial role in cofactor binding and the latter contains ATPases. The topological differences in ADC family were reported by Castillo and coworkers from a structural perspective [37]. The non-outliers have anti-parallel β-sheet with a Greek-key architecture termed as ferredoxin reductase-like barrel (Figure 3a). The outlier domains (ADC) have the topology of double-psi β-barrel structure with a six-stranded β-barrel (for a superimposed view, see Figure S3). The double-psi β-barrel belongs to the most frequently occurring class, but it has a distinctive topology. It consists of two interlocked motifs that are related by a pseudo-twofold axis in which, the parallel strands form two psi-structures [38], [39] (Figure 3b). The topology difference is shown in Figure 3c and d).

### Structural Deviation in Specific Taxa Leads to Different Mode of Substrate Binding

**Figure 4:**
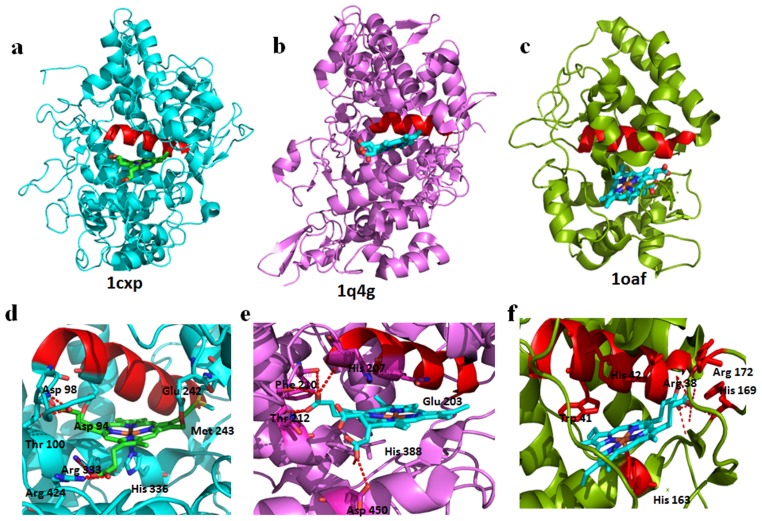
Representative structures of mammalian ((a) MPO (b) PGHS) and (c) non-mammalian peroxidases (1gwu). Both MPO and PGHS are outliers belonging to the mammalian myeloperoxidase family (d)–(f).The peroxidase active site residues and interaction with Heme is shown. In all the cases, the proximal His (H336, H388 and H163) is involved in coordination. Helix H2 and Helix B interact with heme group and are highlighted in red color in all the three structures.

In the current dataset, a single superfamily, of peroxidases, could be observed where high structural variations reside between domains within the superfamily, where the domains are from different taxa. Peroxidases are heme-containing enzymes which use hydrogen peroxide as electron acceptors to catalyse a number of oxidative reactions [40]. Peroxidases are found in almost all the taxonomic classes. On the basis of structural and functional similarity, many peroxidases are added into the heme-dependent peroxidase superfamily. There are a total of eight domains from three different families (CCP-like, catalase-peroxidase KatG and myeloperoxidase-like) in our PASS2 dataset. The CCP-like and catalase-peroxidase KatG families contain domains of plant, fungi and bacterial peroxidases which align well with low RMSD (Figure S4). However, the animal peroxidases that belong to the myeloperoxidase-like family exhibit structural variations. These two highly deviant domains, the outliers, are myeloperoxidase (MPO; PDB ID: 1cxp) and prostaglandin H2 synthase (PGHS; PDB ID: 1q4g). It is already known that myeloperoxidase and C-terminal domain of prostaglandin H2 synthase are homologous to each other (for the superimposed view, please see Figure S4). Although the members retain equivalent helices across the families, the structural elaborations and differences in the arrangement of the secondary structure elements are the major cause for these two members to appear as outliers in a family-specific manner (Figure 4a&b). Figure 4c shows ascorbate peroxidase from soybean (PDB ID: 1oaf) to represent all the non-outliers. Apart from the structural differences, an interesting difference in the substrate-binding pattern is also observed between mammalian and non-mammalian peroxidases [41]. The orientation of the heme is similar, where the propionic groups point towards the amino-terminus of helix H2 in both mammalian peroxidases MPO and PGHS (Figure 4a & 4b), while the orientation is opposite in non-mammalian peroxidases. The propionic groups point towards the carboxy terminus of the equivalent B-helix in non-mammalian peroxidases (Figure 4c) [42]. The overall similar topology and function also suggests that these two domains would have evolved from a common ancestor. The conserved residues (Thr100 and His336 in MPO and Thr212 and His388 in PGHS) interact with heme in a similar manner [43], [44]. In fact, in all the peroxidases, the coordination of the heme metal by a proximal histidine residue is conserved across the heme-dependent peroxidases and serves to impart a low, negative reduction potential upon the heme iron (Figure 4d–f) [45].

### SCOP Families that Get Separated by Circular Permutation

**Figure 5:**
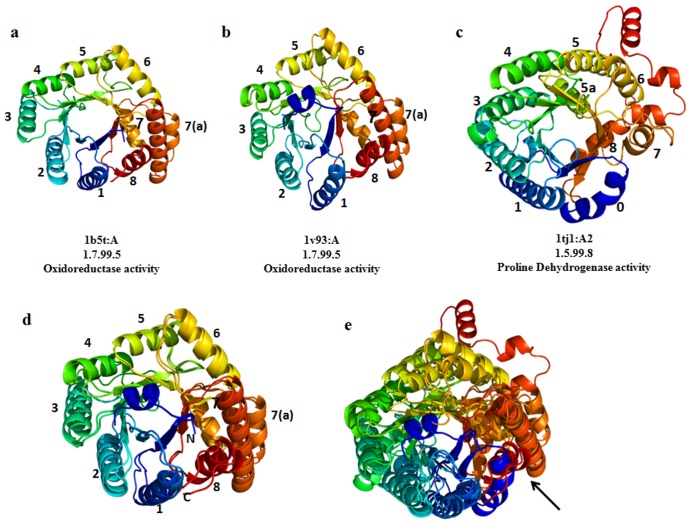
The structural difference between the MTHFR and PutA PRODH families. 1tj1 is the structurally deviant member of the FAD-linked oxidoreductase superfamily. The topology of 1tj1 structure is slightly different from the classic β_8_α_8_ barrel topology and also functionally diverse. The Helix number is also shown in the figure. The pdb ID and the chain ID along with their EC number and enzymatic activity are mentioned. The figures are made in pymol with spectrum coloring which shows N-terminus(blue) to C-terminus(red).(d) The superposed pose of 1b5t and 1v93 (f) Superimposed view of all the three domains (1b5t,1v93 and 1tj1).The N-terminus helix in 1tj1 is aligned with C-terminal helix of the other two domains (shown with an arrow). All other helices are not aligned properly.

There are two out of 41 superfamilies, FAD-linked oxidoreductases and carbohydrate phosphatases, which retain outlier domains due to circular-permuted topology. The FAD-linked oxidoreductase superfamily consists of two families, namely methylenetetrahydrofolate reductase (MTHFR) and proline dehydrogenase domain of bifunctional PutA protein, both sharing a common TIM-barrel fold. Proteins in these two families are the only known structures for FAD cofactors bound to a TIM barrel, the PutA PRODH domain and methylene tetrahydrofolate reductase. One out of three members from the family, proline dehydrogenase exhibits large structural difference which is reflected as high RMSD. The outlier, PutA PRODH barrel, exhibits three deviations from the classic (α/β)_8_ topology (Figure 5). First, the barrel begins with a helix (α0) rather than a strand. Second, there is a helix inserted between α5 and β6 (denoted α5a).This helix is functionally important, since the active site residues are located at its N-terminus. Finally, α8 is located above the barrel rather than being beside it, when viewed down the barrel axis. The location of α8 is also critical for function, as this helix contributes four active site residues. Thus, α8 is critical for PRODH function of PutA. These two families are related by circular permutation of the barrel, such that strands 1–8 of the PutA barrel correspond to strands 8,1–7 of the MTHFR barrel, and α0 of PutA aligns with α7 of MTHFR [46].

This kind of circular permutation problem could be treated by stringent structure alignment protocol, but we might lose the identification of functional differences that occur between families. Apart from circular permutation, the differences in EC numbers confirm that the outliers and non-outliers have different enzymatic function. Helix α8 plays essential roles in PutA’s PRODH function, whereas the corresponding region in MTHFR does not participate directly in binding to substrates or cofactors [47]. The overall structural similarity to the classic TIM barrel fold does not imply similar function here.

### Mechanistically Diverse Enzyme Families can Retain Functional Difference at Domain Linker Regions

**Figure 6:**
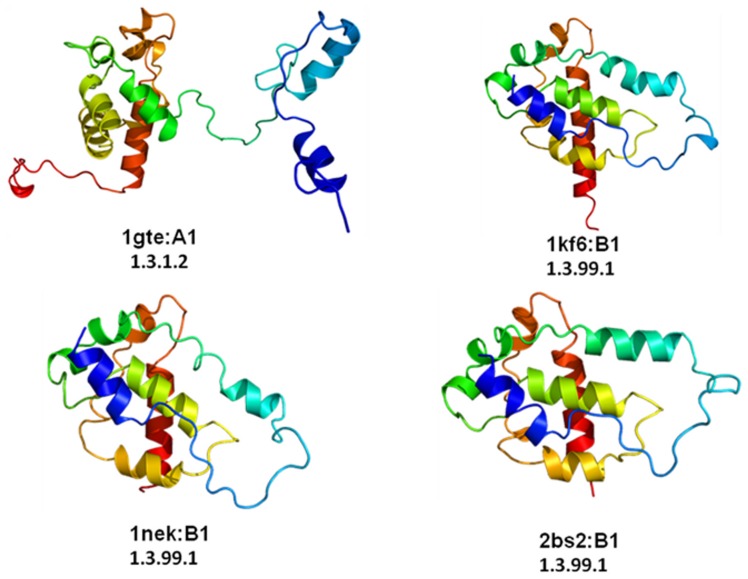
Structural view of the four domains of alpha-helical ferredoxin superfamily. 1gte:A1 is the structurally deviant member of the superfamily with different E.C. number. It has slightly elongated N-terminal tail part. All the other three domains superimpose well with less than 3Å RMSD.

The α-helical ferredoxin superfamily is represented by four domains in the PASS2 database, derived from two different families, namely C-terminal domain of fumarate reductase/succinate dehydogenase iron-sulfur protein and N-terminal domain of dihydropyrimidine dehydrogenase (DPD). The members of the fumarate reductase/succinate dehydrogenase, have high structural conservation and enzymes from *E. coli* can bidirectionally catalyze the interconversion of succinate and fumarate and each can functionally replace the other to support growth [48]. On the other hand, DPD is a cytosolic enzyme catalyzing the NADPH-dependent reduction of uracil and thymine to the corresponding 5, 6-dihydropyrimidines, the first and rate-limiting reaction in the three-step pathway of pyrimidine degradation [49]. Among the four domains of this α-helical ferredoxin superfamily, one domain shows high RMSD (for superimposed view, see Figure S5) belongs to the DPD family. The structural variation is due to an extended N-terminal and a C-terminal linker region which connects the adjacent domain [50] (1gte: A1 in Figure 6). Apart from the structural variation, the difference in the EC number clearly shows that the outlier has different enzymatic function with the remaining domains.The outlier Dihydropyrimidine dehydrogenase (DPD) enzyme is involved in pyrimidine degradation. The other non-outlier domains are involved in oxidation and reduction of succinate and fumarate. In this particular case study, the structural difference of addition of domain linker and extra N-terminal part contributes to the obvious functional diversity, since the outlier is having distinct EC number.

### Difference in the Secondary Structural Elements and Topology of an Outlier Leads to Functional Differences

**Figure 7:**
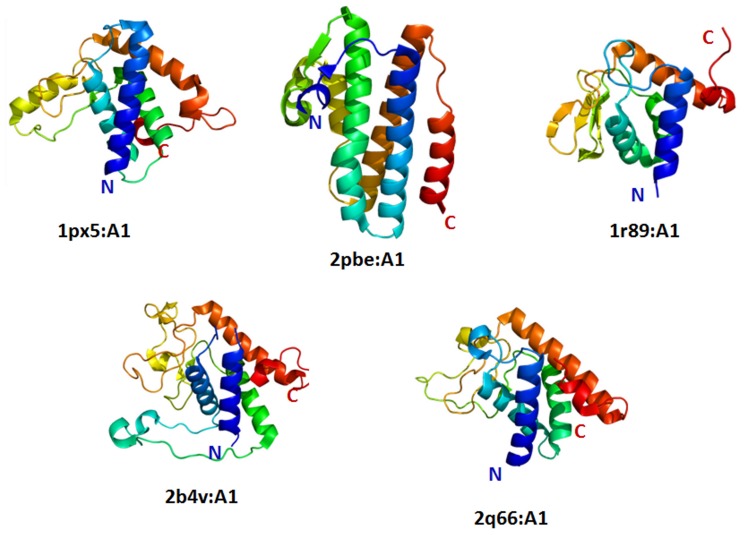
Structural view of all the domains PAP/OAS1 substrate-binding domain superfamily. Among the five domains, 2pbe:A1 is structurally and functionally different member. The architecture of the domain is different from all the other domains.

Within the dataset of 41 superfamilies with structural outliers, there are five superfamilies, PAP/OAS2 substrate binding domains, ParB/Sulfiredoxin, UBA-like, LigT-like and TrpR-like, where the outlier retains difference in secondary structural content and distinct topology. The superfamily of PAP/OAS2 substrate binding domain has five members in the PASS2 database. The superfamily contains domains from families Poly(A) polymerase and 2′-5′-oligoadenylate synthetase. The structural alignment of these five domains revealed that one domain belongs to AadK C-terminal domain-like domain family is structurally different. This Amino glycoside 6-adenylyltransferase (AdaK) domain is a modifying enzyme associated with bacterial resistance by adenylating streptomycin in *Bacillus subtilis* [51], where five helices are arranged as a bundle-like structure (Figure 7, entry 2pbe:A1). In the other four non-outlier domains (entries 1px5:A1, 1r89:A1, 2b4v:A1, 1q66:A1), the helices are not placed parallel to each other (Figure 7). The superimposed view of all the five domains where the structural differences and the secondary structures can be seen in Figure S6).

### Structural Divergence Exist between Swapped and Non-swapped Domains

**Figure 8:**
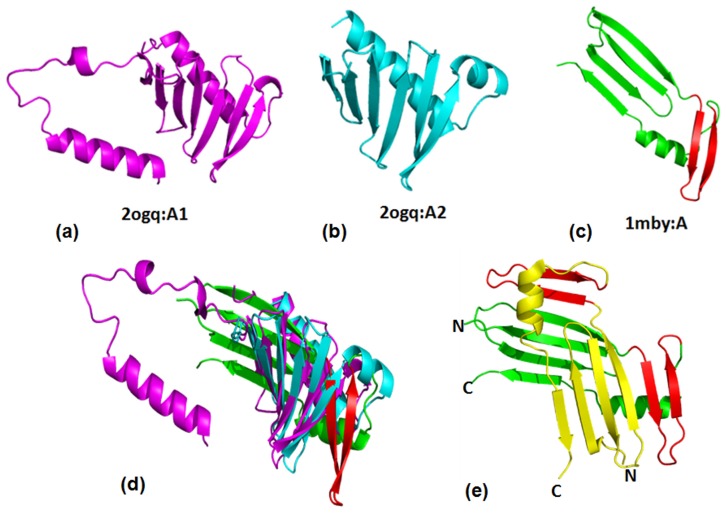
The structural view of members of the polo-box domain superfamily. (a) and (b) are polo-box domain from the family “polo-box duplicated region” (c) 1mby:A is a structurally deviant member of the superfamily which belongs to swapped polo-box domain family.(d) superimposed view of all the three domains shows the alignment is not good. (e) Dimeric form of Polo-box domain in swapped conformation (PDB ID: 1mby). The swapped part is highlighted in red.

Domain swapping is an important phenomenon involved in many biological processes such as in protein molecular evolution, functional regulation and in the formation of protein conformational/deposition diseases, such as amyloid and prion diseases [52]. Many structure alignment protocols attempt to circumvent problem of aligning domain swapped examples by attributing global similarities between the swapped and non-swapped protein domains. However, we observed that the domain swapped entry exists as a structurally deviant member of the superfamily (Figure 8). The superfamily Polo-box domain consists of β(6)-α motif arrangement, where all the six β sheets are anti-parallel. Members of this superfamily are protein kinases which are of important regulators in diverse aspects of the cell cycle and cell proliferation [53]. This superfamily consists of two families namely ‘Polo-box duplicated region’ and ‘Swapped Polo-box domain’. The former family consists of duplicated two polo-box domains (Figure 8a & b). The second family contains one member (1mby:A) which forms a swapped polo-box domain dimer. The crystal structure (PDB ID:1mby) of the polo domain is a swapped dimer with two α-helices and two six stranded β-sheets [53].The topology of the 1mby:A has an extended strand segment, from its N- to C-terminus five β-strands (1–5), one helix and C-terminal β-strand (Figure 8c). β-strands 6, 1, 2 and 3 from one subunit form a contiguous antiparallel β-sheet with β-strands 4 and 5 from the second subunit (Figure 8e).

The ‘polo-box duplication region’ family has two domains arising from the same protein chain. The outlier is a domain-swapped polo-box. It is already reported that the polo domains form dimers both *in vitro* and in a crystal environment, self-associates *in vivo* and localizes to mitotic structures. The conservation of the hydrophobic core and dimer interface residues, the presence of two copies of the polo domain in most Polo-like kinases and the covariance across tandem polo domains in most Plks suggest that the ability to adopt a dimeric conformation may be a general characteristic feature of all polo domains and that domain swapping may occur in an intramolecular manner for some family members [54]. There are three other superfamilies such as Ribosomal protein L25-like, C-type lectin-like, Prokaryotic SH3-related domain, where some members undergo swapping of domain or segment which leads to structural differences.

### N- and C-terminal Extensions could be Required for Diversity in Overall Biological Function

**Figure 9:**
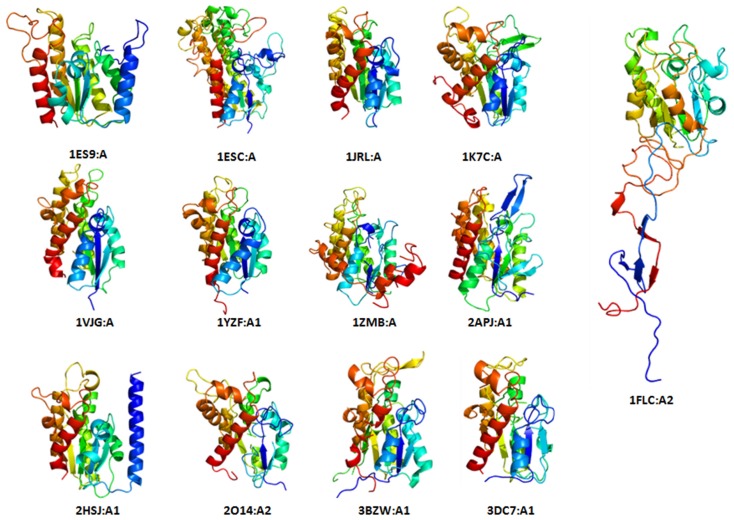
Structural view of all the distantly related domains of SGNH hydrolase superfamily. Conformations of N- and C-terminal part are conserved in all domains except 1FLC:A2. This domain has extra elongated N and C-terminal part which is involved in fusion.

We noticed that N-terminal and C-terminal embellishments of outliers occur as insertions and could meaningfully add functional variety within superfamilies. There are seven superfamilies that come under this group, namely, SGNH hydrolases, alkaline phosphatase-like domains, PAP/Archaeal CCA-adding enzyme (C-terminal domain), GAF-like domains CYTH-like phosphatases, GatB/YqeY motif and Sialidases. Apart from this, there are two superfamilies, heme-dependent catalase-like, bacterial luciferase-like which have domains with incomplete core structures due to deletion events. SGNH hydrolase superfamily has 13 protein domains in the PASS2 database and they have similar fold to flavoproteins, namely a three-layer α/β/α structure, where the β-sheets are composed of five parallel strands. The superimposed view of all the domains is shown in Figure S7a. Among them, an outlier, the esterase domain of haemagglutinin-esterase-fusion glycoprotein HEF1 domain, retains N- and C-terminal embellishments (Figure 9). These structural elaborations could lead to structural divergence resulting in more profound structural changes, making it harder to recognize the core similarity with other protein domains in the superfamily. The haemagglutinin-esterase glycoprotein monomer consists of three domains: an elongated stem active in membrane fusion, an esterase domain, and a receptor-binding domain, where the stem and receptor-binding domains together resemble influenza A virus haemagglutinin. The esterase domain belongs to this SGNH hydrolase superfamily and contains non-contiguous sequence: the receptor-binding haemagglutinin domain is inserted into a surface loop of the esterase domain and the esterase domain is inserted into a surface loop of the haemagglutinin stem (Figure S7b). N-terminal (F1) and C-terminal (F2) regions participate in membrane fusion, either by controlling the low-pH-induced conformational change required for fusion or during the formation of a fusion pore [55].

### Outlier with Internal Repeat or Duplication could Retain Discrete Function

**Figure 10:**
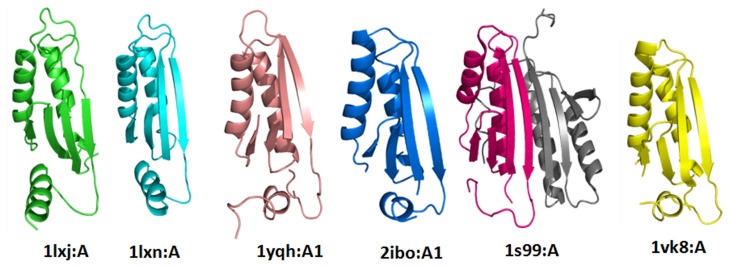
Structural view of members of MTH1187/YkoF-like superfamily. The domain1S99:A has internal repeat of βαβ fold highlighted in grey colour.

In the current analysis, two superfamilies (MTH1187/YkoF-like, EPT/RTPC-like superfamiles) retain outliers that acquire internal repeats or clear duplication of the domain fold, and therefore acquire difference in biological function. MTH1187/YkoF-like superfamily consists of six members from two families. The family MTH1187-like contains only hypothetical protein domains and the family putative thiamin/HMP-binding protein YkoF has putative thiamin/HMP-binding protein domains. Thiamin/HMP-binding protein domain is involved in the hydroxymethyl pyrimidine (HMP) salvage pathway and the other family (MTH1187-like members) contains domains of unknown function. The superfamily members retain a ferredoxin-like fold with α+β barrel with anti-parallel β-sheet topology (Figure 10).The superimposed view of the domains is shown in Figure S8. All the six members are similar in their architecture and topology, but one domain (1S99:A) which belongs to putative thiamin/HMP-binding protein family has internal tandem repeat of a ferredoxin-like βαββαβ fold. Each of the repeats has similarity with other family MTH1187-like members. The outlier domain has eight-stranded, anti-parallel β-sheet, with the strands arranged in the order 23148576. The four connecting α-helices are stacked against one face of the β-sheet, leaving the other side exposed. The two ferredoxin-like motifs form a side-to-side contiguous β-sheet *via* an anti-parallel interaction between β-strands 4 and 8 [56]. The superfamily EPT/RTPC-like has an outlier domain which is in non-duplicated structure, where other domains are in the duplicated form.

## Conclusion

**Figure 11:**
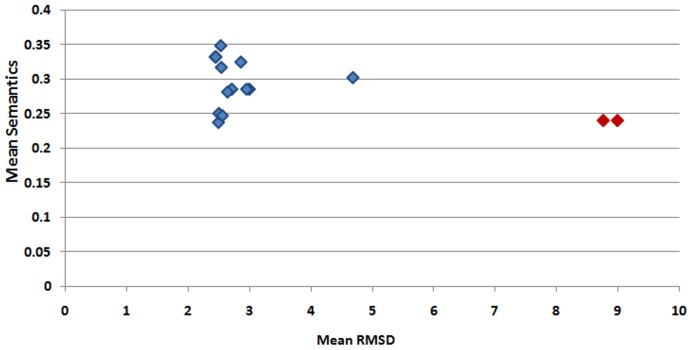
Comparison of mean structural deviation (rmsd) of the members (shown in X-axis) and mean GO semantics scores for ADC-like superfamily (shown in Y-axis). Higher RMSD reflects higher structural deviation and lower mean GO semantics shows lower functional correspondence of a member with other members of that superfamily. The points corresponding to outliers are shown in red colour and non-outlier members of a superfamily are marked with blue colour.

As described above, changes accumulated in a protein structure, are being used by the living machinery for related but slightly different biological functions, contributing to a general evolutionary pressure to preserve these structural changes. The paper explains that structure-based sequence alignment methods are reliable for the identification of structural variations within a superfamily. All the family-specific outliers from 41 superfamilies have been examined critically. We have observed that major structural variations occur due to differences in the structural topology, domain swapping, circular mutation, irregular elaborations, duplication and insertion. High structural variations within domains of the same superfamily could be accompanied by functional differences. A quantitative comparison of Gene Ontology terms of functional characterization (as described in Methods) shows that structural variations are accompanied by diversity in protein function (please see Figure 11 for ADC-like superfamily and http://caps.ncbs.res.in/download/pass2_outliers/GO_semantics for similar plots for the other superfamilies discussed). Outliers exhibit lower GO semantics similarity scores with the other members of the superfamily in comparison to the other members with each other.

Protein structure evolution seems to be initiated from these subtle changes giving rise to functional variety and gradually add up to a new fold itself which would challenge fold prediction methods and extrapolation of function. These examples are implications for the need for reliable structural classification schemes. This approach of looking at protein structure alignments at a superfamily level provided us a vast understanding of the similarities and deviations among the members pointing towards their subtle differences in functions. The observations discussed here hint that functional characterization by mere structure conservation will be an over-simplified assumption. *Albeit* the knowledge of fold-level similarities and superfolds, all these data further emphasize that functional similarities cannot be extrapolated from mere structural conservation. A detailed study of these differences can provide a better picture of different protein architecture from an evolutionary perspective. The mutations responsible for these structural changes can be of extreme importance to understand the protein folding chemistry at an amino acid granularity.

## Supporting Information

Figure S1 **Flowchart explaining the types of outliers.** The total number of superfamilies having outliers and the types of outliers such as structural, functional, subgrouping of outliers.
(TIF)

Figure S2 **Lipocalin superfamily (50814) outliers subgroups.** This superfamily has total of 14 outliers and interestingly they forms subgroups among themselves. RMSD based phylogeny and their subgroups superposition are shown**.**
(TIF)

Figure S3 **Superimposed view of ADC-like superfamily.** (a) Superimposed view of all the 16 domains of ADC-like superfamily. (b) Superposed figure of all the non-outliers. (c) Superposed view of two outliers.
(TIF)

Figure S4 **Superimposed view of domains of Heme-dependent peroxidases superfamily.** (a) Superimposed view of outliers (1cxp:C,D 1q4g:A1).The helix is highlighted in red. (b) Superimposed view of all the non-outliers. They superimpose with low RMSD.
(TIF)

Figure S5 **Superimposed view of all domains of Alpha-helical ferredoxin (46548) superfamily.** The N-terminus to C-terminus is coloured from Blue to Red.
(TIF)

Figure S6 **Superimposed view of all the domains of PAP/OAS1 substrate-binding domain (81631) superfamily.** The outlier is shown in pink colour.
(TIF)

Figure S7 **The superimposed view of all the members of SGNH hydrolase (52266) superfamily.** All the non-outliers are coloured by pymol spectrum colouring and the outlier is in pink. (b) The structure of haemagglutinin-esterase glycoprotein monomer.
(TIF)

Figure S8 **Superimposed view of all the members of MTH1187/YkoF-like (89957) superfamily.** The outlier is highlighted in pink colour.
(TIF)
