# Supplementary figures and images for: Rebelling for a Reason: Protein Structural “Outliers”
Source: PLoS One. 2013 Sep 20;8(9):e74416. doi: 10.1371/journal.pone.0074416 (PMC3779223; doi:10.1371/journal.pone.0074416)

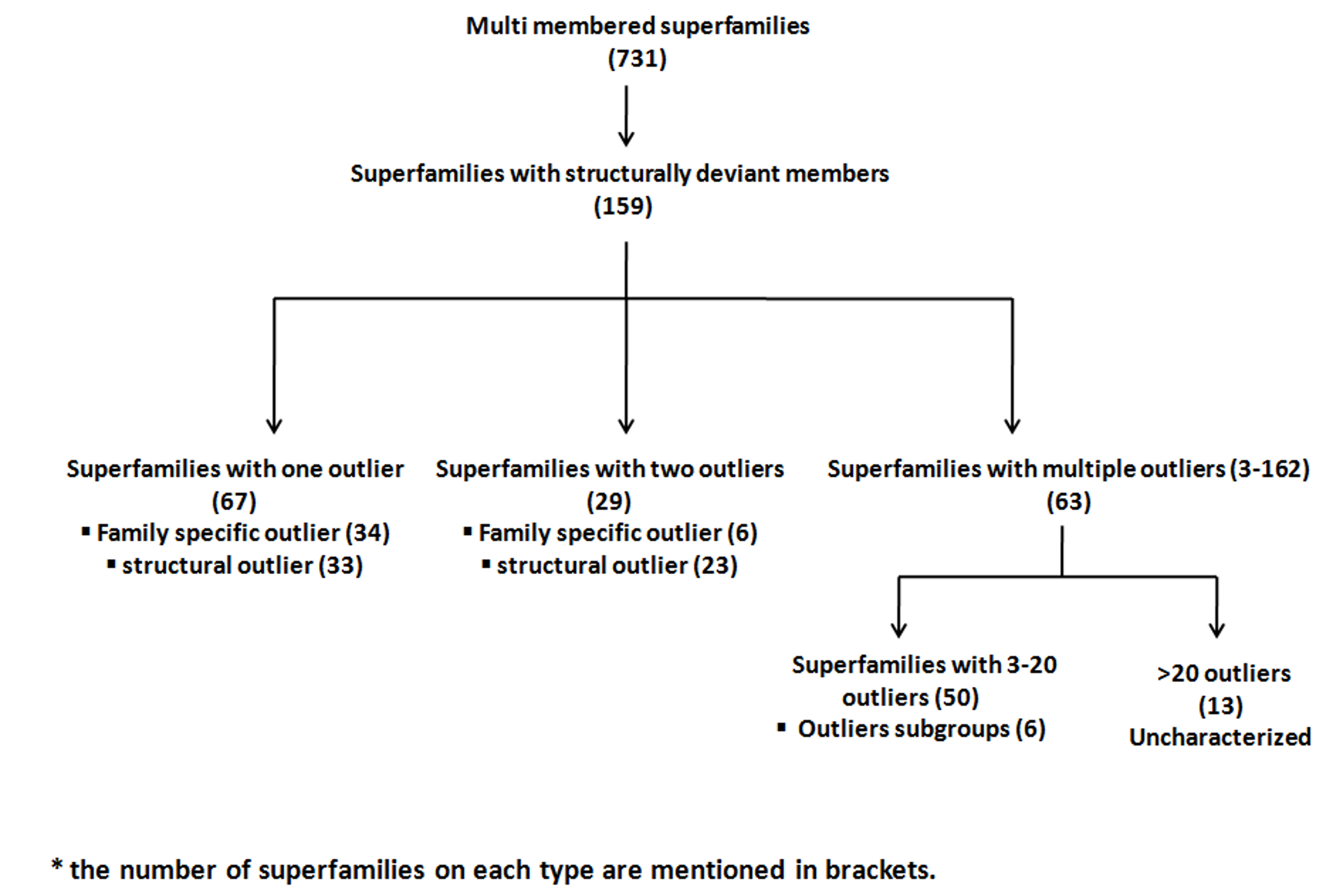

Supplement: Figure S1 — Flowchart explaining the types of outliers. The total number of superfamilies having outliers and the types of outliers such as structural, functional, subgrouping of outliers. (TIF) [file pone.0074416.s001.tif]

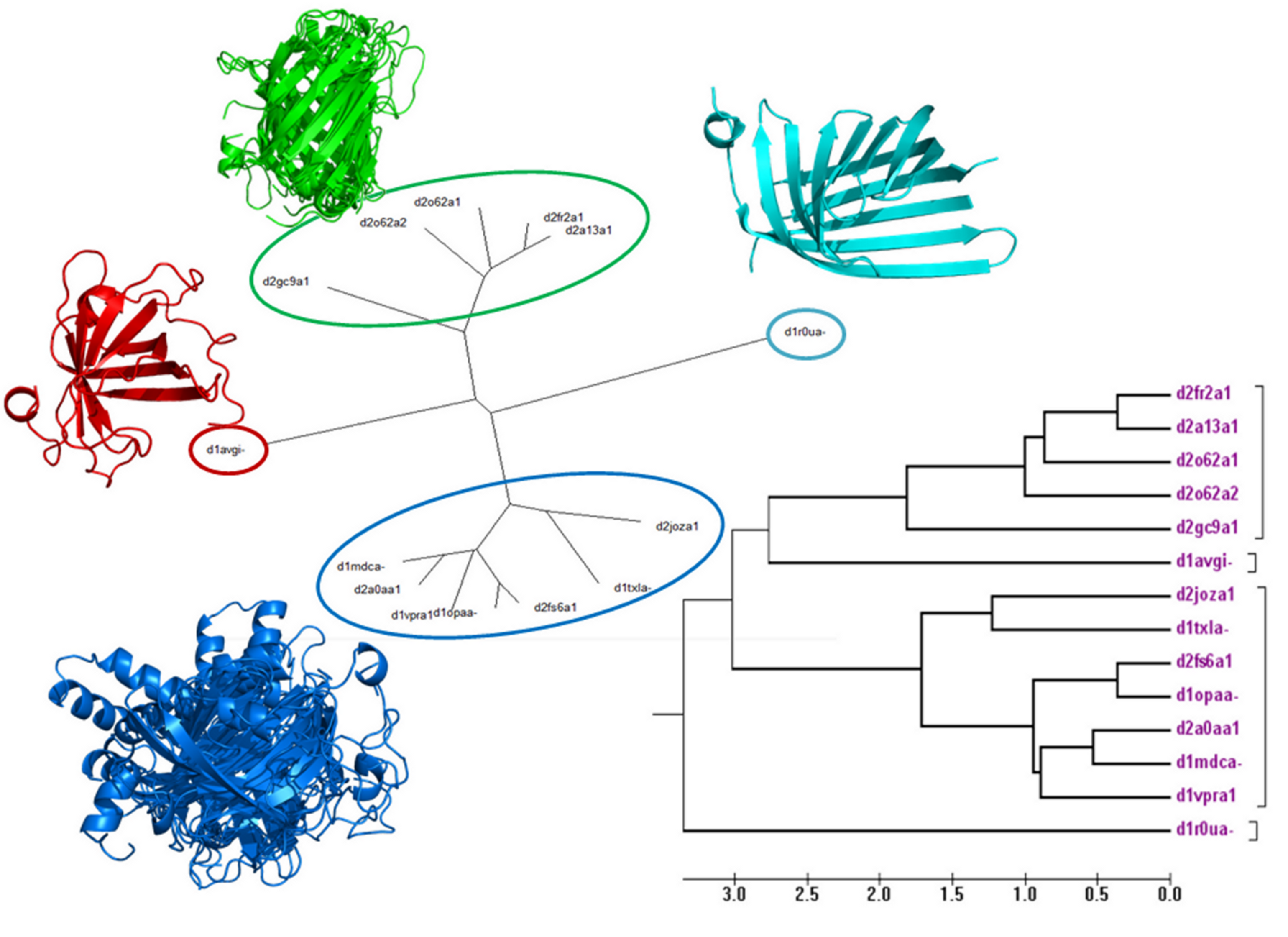

Supplement: Figure S2 — Lipocalin superfamily (50814) outliers subgroups. This superfamily has total of 14 outliers and interestingly they forms subgroups among themselves. RMSD based phylogeny and their subgroups superposition are shown. (TIF) [file pone.0074416.s002.tif]

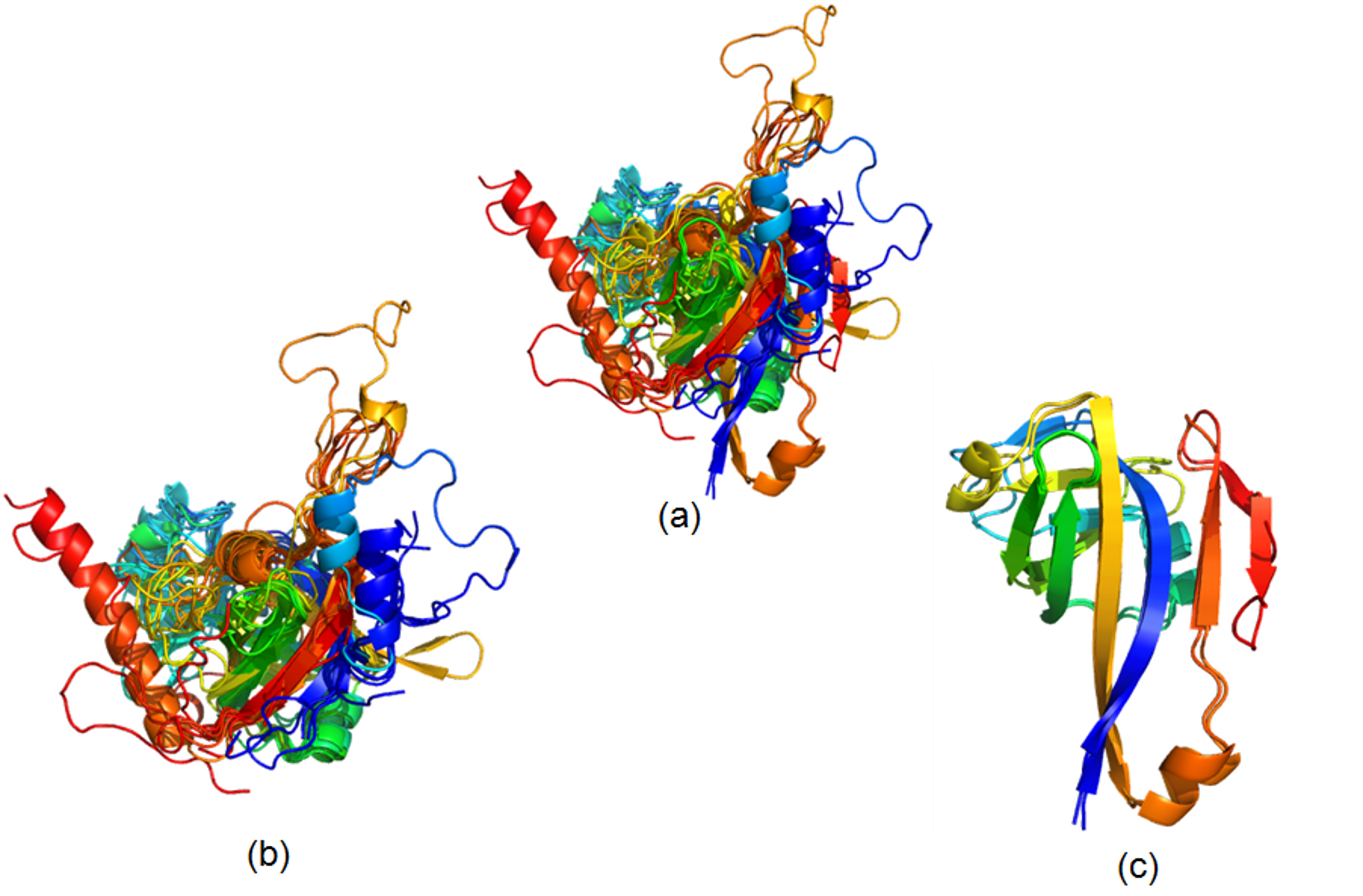

Supplement: Figure S3 — Superimposed view of ADC-like superfamily. (a) Superimposed view of all the 16 domains of ADC-like superfamily. (b) Superposed figure of all the non-outliers. (c) Superposed view of two outliers. (TIF) [file pone.0074416.s003.tif]

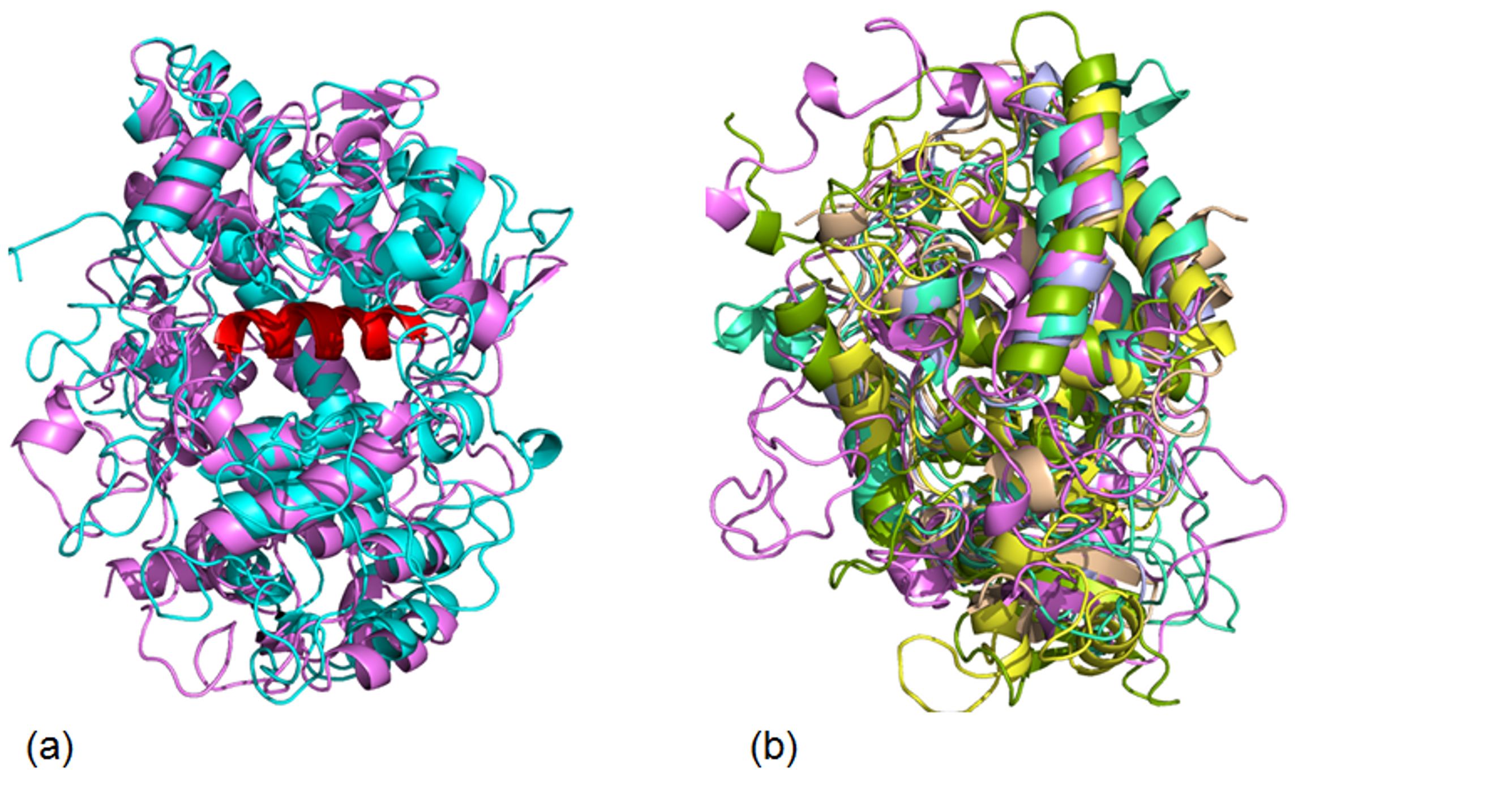

Supplement: Figure S4 — Superimposed view of domains of Heme-dependent peroxidases superfamily. (a) Superimposed view of outliers (1cxp:C,D 1q4g:A1).The helix is highlighted in red. (b) Superimposed view of all the non-outliers. They superimpose with low RMSD. (TIF) [file pone.0074416.s004.tif]

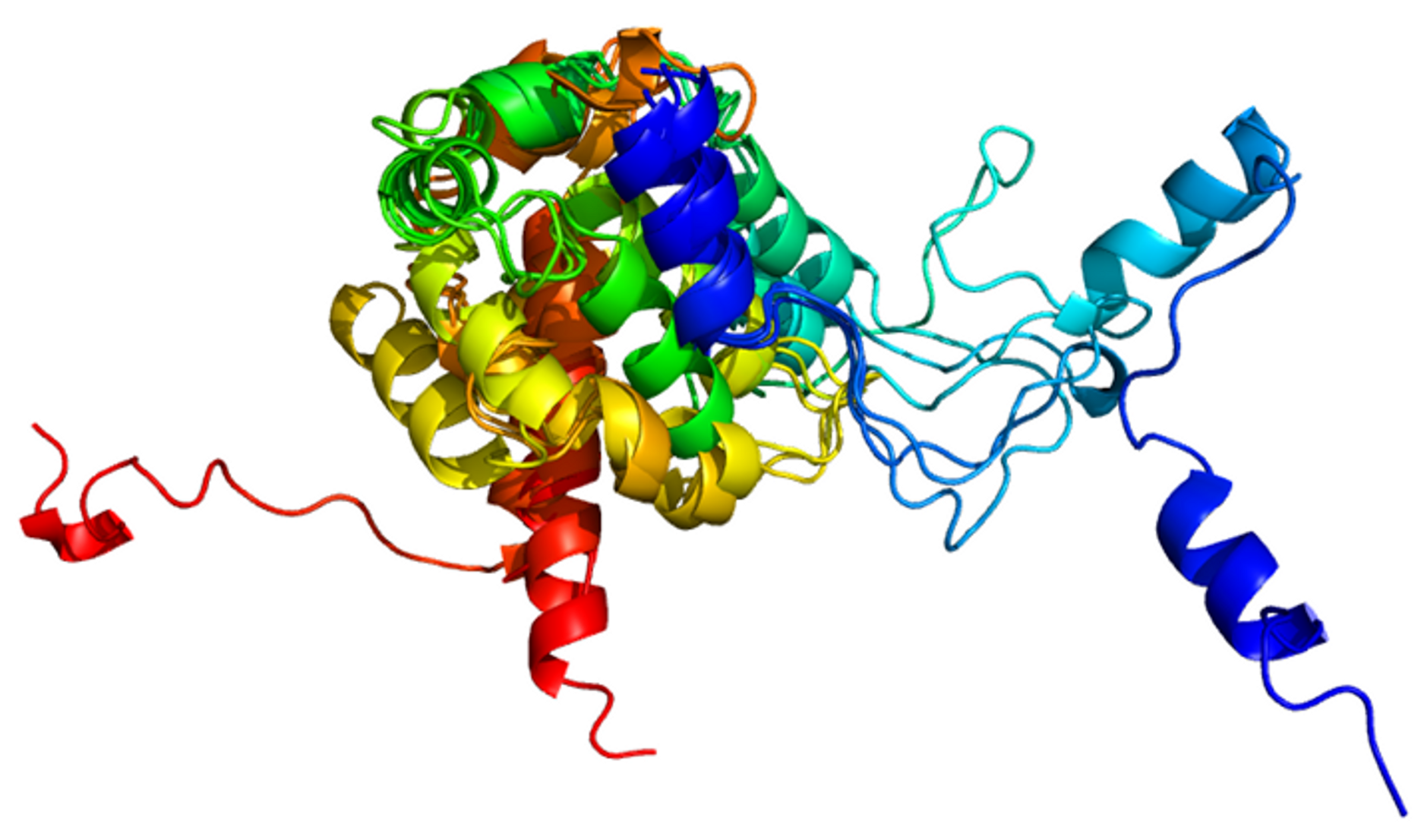

Supplement: Figure S5 — Superimposed view of all domains of Alpha-helical ferredoxin (46548) superfamily. The N-terminus to C-terminus is coloured from Blue to Red. (TIF) [file pone.0074416.s005.tif]

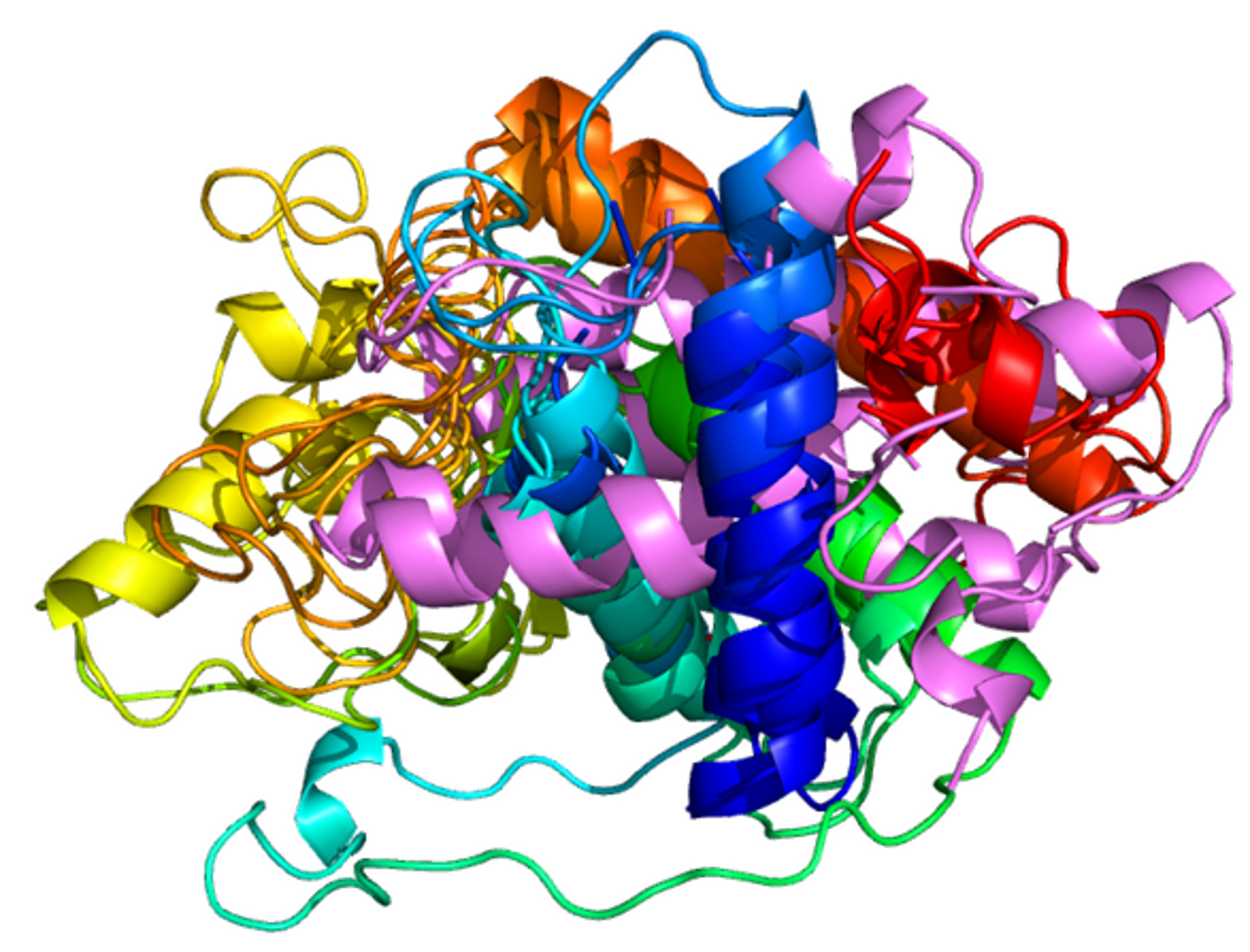

Supplement: Figure S6 — Superimposed view of all the domains of PAP/OAS1 substrate-binding domain (81631) superfamily. The outlier is shown in pink colour. (TIF) [file pone.0074416.s006.tif]

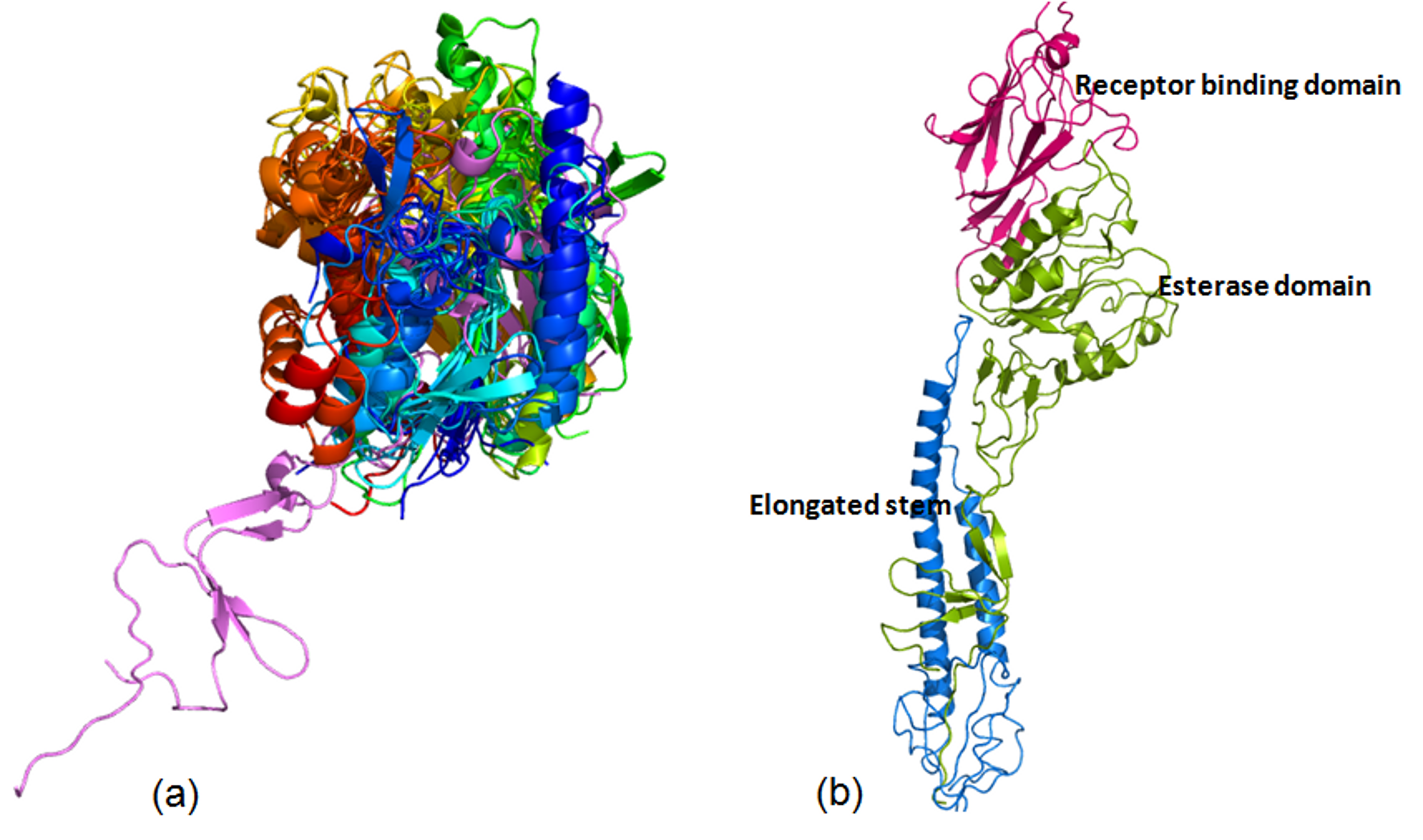

Supplement: Figure S7 — The superimposed view of all the members of SGNH hydrolase (52266) superfamily. All the non-outliers are coloured by pymol spectrum colouring and the outlier is in pink. (b) The structure of haemagglutinin-esterase glycoprotein monomer. (TIF) [file pone.0074416.s007.tif]

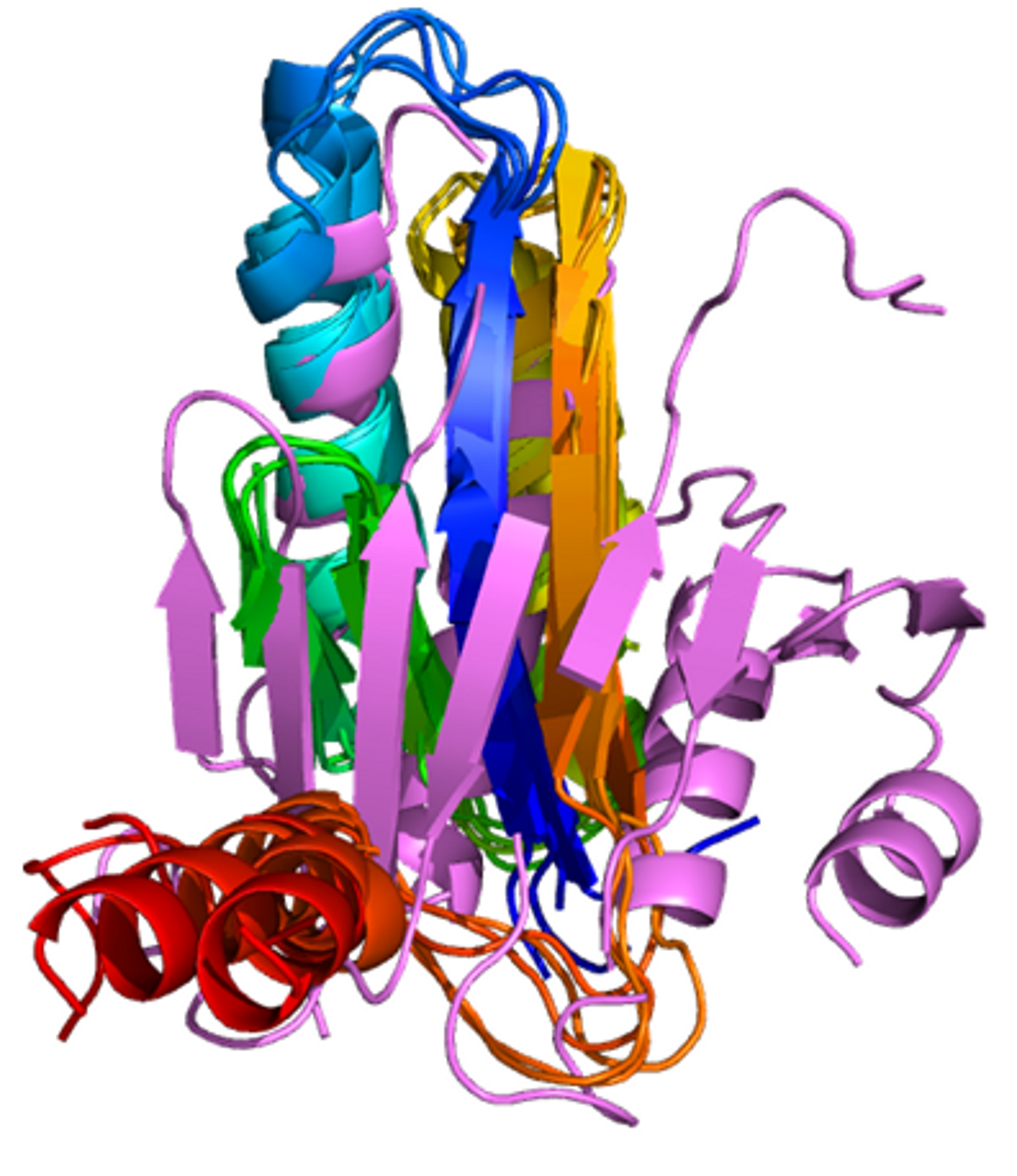

Supplement: Figure S8 — Superimposed view of all the members of MTH1187/YkoF-like (89957) superfamily. The outlier is highlighted in pink colour. (TIF) [file pone.0074416.s008.tif]
